# Subcutaneous EEG Monitoring Reveals AED Response and Breakthrough Seizures

**DOI:** 10.1155/2020/8756917

**Published:** 2020-01-28

**Authors:** Sigge Weisdorf, Ivan C. Zibrandtsen, Troels W. Kjaer

**Affiliations:** ^1^Zealand University Hospital, Department of Neurology, Roskilde, Denmark; ^2^University of Copenhagen, Department of Clinical Medicine, Copenhagen, Denmark

## Abstract

Unrecognized seizures are a common problem in temporal lobe epilepsy potentially leading to undertreatment. Objective seizure counting using EEG home monitoring for prolonged periods with a minimally invasive device has not been feasible until now. We present a case in which a novel, subcutaneous EEG device was utilized to provide an objective seizure count. This information revealed unrecognized breakthrough seizures and informed treatment response, prompting treatment adjustment. The case illustrates how objective seizure counting in epilepsy using new devices can completely change diagnosis and management.

## 1. Introduction

For patients with epilepsy, self-monitoring of disease activity most commonly relies on a seizure diary. Seizure diaries mostly depend on input from the patient and therefore cannot account for any unrecognized seizures. Unrecognized seizures are a common problem in temporal lobe epilepsy (TLE) [1] and can obscure the true effect of antiepileptic drugs (AEDs). Objective seizure counting based on electroencephalography (EEG) can reveal unrecognized seizures, but until now, such monitoring has usually been performed during hospital admission, which is impractical and expensive, and can therefore only be performed for a limited time. A novel subcutaneous EEG device has been developed for EEG home monitoring. In a previously published proof-of-concept paper, we showed that the new device records clinically relevant potentials reliably compared to scalp EEG [2]. Here, we present a case in which EEG home monitoring reveals essential information on breakthrough seizures and AED response that would otherwise have remained unknown.

## 2. Case Presentation

A 42-year-old woman known with left-sided temporal lobe epilepsy (TLE) and seizure onset at the age of 18 was referred to the outpatient epilepsy clinic due to recurrence of seizures after two decades of seizure-free remission. She lived with her husband, who reported 1-2 seizures/month, but most of these seizures went unrecognized by herself. In addition, she frequently felt unrefreshed after sleep, possibly indicating that unrecognized seizures also occurred at night. Clinically, three different epileptic seizure types were described:

1. Focal onset seizure with severely impaired awareness. Behavioral arrest, frequently accompanied by manual automatisms. Duration 30–60 seconds.
2. Focal onset seizure with only slightly impaired awareness. Behavioral slowing, but not arrest. Inappropriate behavior.
3. Type 1 or 2 seizure with progression to bilateral tonic-clonic seizure (BTCS).

In addition to her seizures, the patient also reported memory difficulties of increasing severity.

Brain MRI (1, 5T) showed an old lacunar infarct (1 cm^3^) in the anterior part of the left external capsule but no abnormalities in the temporal regions. A previous EEG recorded in her youth suggested an epileptic focus in the left temporal region. Due to the suspicion of unrecognized seizures, she underwent repeated short admissions (up to four days) in an epilepsy monitoring unit (EMU). These investigations confirmed the left temporal epileptic focus and revealed that unrecognized seizures occurred.

Treatment with valproate, levetiracetam, and lacosamide was apparently without major effect on seizure frequency or severity.

A year later, the patient was enrolled in a research study testing a novel subcutaneous EEG device. At this point, she was treated with lacosamide (200 mg × 2) and add-on treatment with lamotrigine was commenced during the study. Written informed consent was obtained from the patient prior to inclusion to the study. The study was approved by the regional ethical committee.

**Figure 1:**
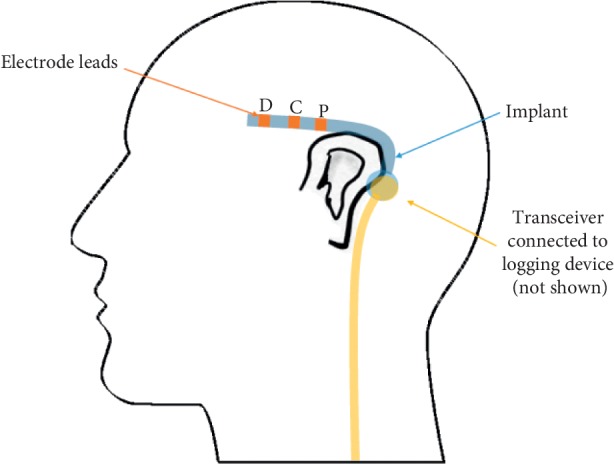
An illustration of the approximate position of the implanted electrode (blue) with the individual leads highlighted. The transceiver from the external logging device (orange) must be attached to the skin near the implanted electrode using double-sided glue pads.

The subcutaneous EEG device, described in more details here [2], consists of two main parts, the implanted electrode and an external logging device for battery and data logging. The electrode and external logging device communicate via a transceiver at either device. The subcutaneous electrode has three leads for recording (a proximal (P), a distal (D), and a reference (R) in between), providing two bipolar channels (P-R; D-R). It was implanted over the left temporal region in local anesthesia through a small incision behind the ear without complications. After implantation there was some mild self-limiting tenderness. The proximal electrode was positioned approximately at T7 and the distal electrode approximately at F7. The approximate position of the implanted electrode is illustrated in Figure 1.

**Figure 2:**
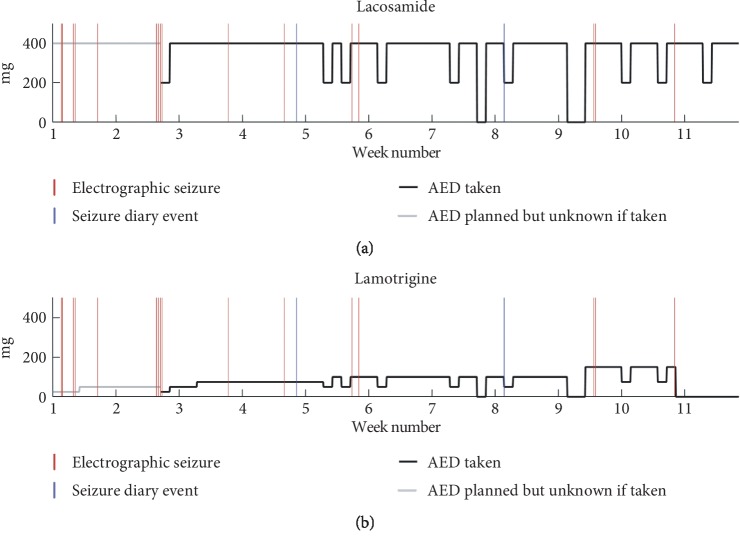
Overview of the monitoring period AED dosages plotted over time for lacosamide (a) and lamotrigine (b). Solid black line is the daily dose taken. It can drop 50% or 100% on some days indicating one or two missed doses. It is greyed out when no information about the dose taken was available. Red vertical lines indicate electrographical seizure. The two first seizures were focal to bilateral tonic-clonic seizures. Blue vertical lines indicate a patient event diary entry. These two do not overlap with any of the electroencephalographic seizures.

The patient had the EEG device active for 76 days during which the actual usage was 63%. All recordings from the subcutaneous electrode was analyzed by an expert visual review of spectrograms using Nicolet Reader to identify electrographical seizures and these were compared to self-reported seizures from the patient's seizure diary. Candidate spectrographical seizure patterns were double-checked by an additional review in the time domain by a board-certified neurophysiologist. Due to the documented seizure unawareness, we regarded the electrographical seizures as the ground truth in this comparison. As such, any self-reported seizures without a corresponding electrographical seizure would be considered a false report.  Figure 2 compares timing of self-reported and electrographic seizures and visualizes AED intake.

The EEG home monitoring revealed 16 electrographical seizures with three different seizure patterns well in line with the reported seizure types. None of the electrographical seizures were reported by the patient, including the seizures progressing to bilateral tonic-clonic seizures (BTCS). However, she reported two events with no corresponding electrographical seizures.

## 3. Discussion

The case exemplifies a situation where there is an important discrepancy between self-reported seizure counts and objective seizure counts, where the pattern of self-reported counts lead to an interpretation of inefficacy of lamotrigine whereas the objective seizure counts suggest a positive effect of lamotrigine add-on therapy.  Figure 2 shows that the frequency of electrographical seizures seems to decrease as the dose of lamotrigine is increased. This hypothesis is weakened by the fact that only a single seizure occurs after permanent discontinuation of lamotrigine, but only approximately one week off lamotrigine was monitored. Regardless of the efficacy of add-on lamotrigine, the possible effect is not evident from the self-reported seizures at all. This case study highlights the pitfalls of basing decisions on self-reported information. With objective seizure quantification, a completely different picture of response to medication emerges that would otherwise have remained hidden. This suggests important advantages of ultralong-term monitoring for objective seizure counting in the management of epilepsy.

Figure 2 also informs on the relation between missed AED doses and breakthrough seizures. Qualitatively, there seems to be a tendency towards breakthrough seizures within a few days of a missed dose. Our dataset does not allow for proper testing of this hypothesis. Nonetheless, the data demonstrate that the continuous EEG monitoring enables such investigations, given enough events. However, it is unsurprising that missed AED doses could precipitate breakthrough seizures. The subcutaneous EEG device, however, offers a quantification of the problem based on an objective seizure count, making the results more reliable and reproducible.

**Figure 3:**
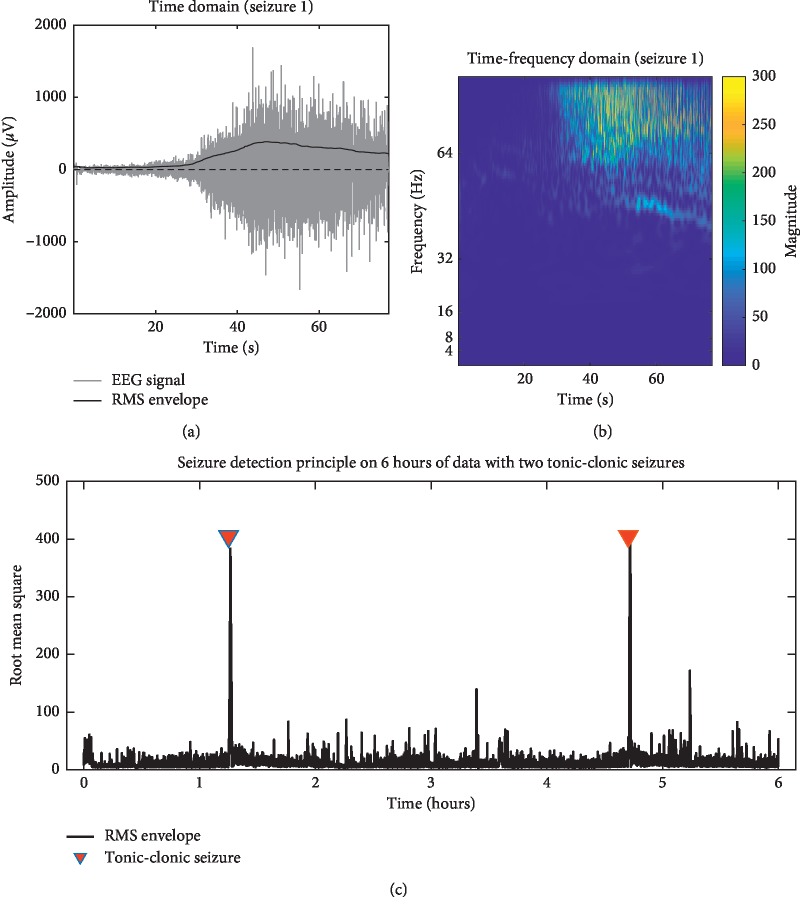
Detection of BTCS. (a) shows 80 seconds of a BTCS in the time domain. Superpositioned on the EEG signal, the bold black line shows the root mean square (RMS) envelope, which increases during the tonic-clonic phase of the seizure. (b) shows the time-frequency decomposition from a continuous wavelet transform of the same seizure. During the tonic-clonic phase, a high-frequency component can be observed, approaching the Nyquist frequency of the device (103.5 Hz). (c) shows the RMS envelope of a six-hour epoch containing two BTCS indicated with red triangles. The RMS shows an abrupt increase only in close relation to these seizures, providing proof-of-concept that the device can be used for the detection of BTCS.

The detection of BTCS is of particular interest to physicians and patients alike, as these seizures have the highest risk of secondary injuries and sudden unexplained death in epilepsy (SUDEP) [3] and a greater impact on life quality [4]. These factors often warrant more aggressive treatment of BTCS. Our dataset contained two BTCS. We used a slightly modified version of a method previously described [5] to investigate if the subcutaneous EEG recording contains enough seizure-related EMG activity to form the basis for a simple tonic-clonic seizure detection algorithm. We calculated the root mean square (RMS) within 4-second windows across a six-hour recording containing the two BTCS (Figure 3). Cooccurrence of the RMS peaks with the BTCS events demonstrates that it was possible to identify BTCS using this method using simple thresholding. Tonic-clonic seizures, presumably regardless of focal or generalized onset, have very stereotypical and specific signatures in the time domain as has been shown in previous studies [6]. As we observe a similar signature, we can reasonably infer that the seizure event has a tonic-clonic component.

This is a promising basis for the use of the subcutaneous electrode for BTCS detection. A reliable differentiation between focal seizures and BTCS is an important piece of clinical information.

**Figure 4:**
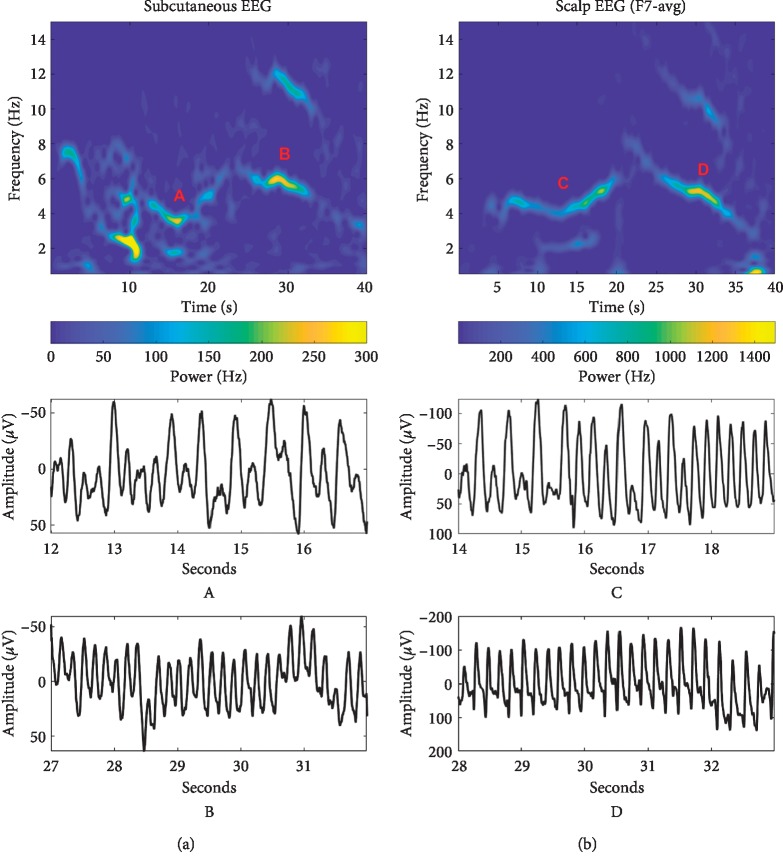
Persistent seizure signature. Comparison of two focal seizures recorded with one year in between from subcutaneous EEG (a) and scalp EEG (b). Time-frequency-power plots of 40 seconds are shown in the top. We see the same overall frequency dynamics between the two, but the subcutaneous device records with lower power and the seizure has more mixed frequencies in the early part. For each seizure, the letters designate different time-domain magnifications shown below for approximately the same corresponding part of the spectrographical signature. At around 15 seconds, 4-5 Hz activity can be observed. Around 30 seconds, 5-6 Hz activity with a sharper morphology in the time-domain is visible, mirrored by the higher-frequency subharmonic visible in this part of the spectrum.

Temporal lobe epilepsy (TLE) is the most common type of focal onset epilepsies in adults [7]. Typical temporal lobe seizure semiology includes speech arrest, impaired awareness, automatisms, and a wide array of other features [8]. Impaired awareness during seizures increases the risk of unrecognized seizure and resulting under-reporting. This may lead to insufficient treatment and risk of additional seizures. Impaired awareness is frequent in temporal lobe epilepsy [1, 9], so while there is nothing unusual about our patient's problem, the solution is extraordinary. Using ultralong-term EEG home monitoring we have provided an example on the advantage of objective vs. subjective seizure quantification and how this can inform management. While the EEG monitoring allowed us to record many electrographical seizures, it also holds its own issues. In our single case, the electrographical seizures were identified by an expert visual review, which is prohibitively resource demanding for routine use of such monitoring. Implementation of long-term monitoring in clinical practice will therefore require automated seizure detection algorithms. We propose that the subcutaneous EEG device would be a well-tolerated platform to acquire data for such automated methods, as it offers the ability to obtain large amounts of data from single individuals, providing a solid basis for personalized algorithms. Evidence that seizure signatures appear unaffected by time, AED changes, and recording modality is important for the seizure detection algorithm design strategies. In a paper from 2017, Zibrandtsen et al. [10] proposed that personalized algorithms could take advantage of within subject seizure stereotopy.  Figure 4 shows a comparison between two seizures recorded from the patient. One was recorded with the subcutaneous electrode and the other was recorded with scalp EEG one year before. A distinct spectrographical pattern (a “signature”) is clearly present in both recordings.

For now, identification of focal seizures relies on an expert review of the data. In the future, this will be assisted by a software solution to identify rhythmic patterns for manual review, significantly decreasing the time needed to review ultralong-term recordings.

The novel device presented in this case does have some limitations. The limited spatial coverage resulting from the small size could lead to seizures from a contralateral seizure focus going undetected, resulting in false negatives. Due to the proximity of the device to the temporal muscle, false negatives could also occur in situations with high muscle activity, e.g., in hyperkinetic seizures. Such activity would cause EMG noise, possibly obscuring electrographical seizure patterns. Rhythmic noise and artifacts, whatever the cause, generally do not look like seizures, so overinterpretation resulting in false positives is less likely, if the reviewer is experienced. Video monitoring would have been helpful in revealing cases of false negative and/or positive seizures, but continuous video monitoring during daily life is neither achievable nor ethical.

In conclusion, this clinical case presents a scenario where the subcutaneous EEG provided a completely different picture of responsiveness to medication compared to seizure diaries, which could lead to a different conclusion about efficacy of add-on AED therapy. A wider use of this technology in clinical practice depends on the development of accurate seizure detection algorithms. We expect that this device and others like it will play a growing role in future epilepsy management.
